# Efficacy of tumor treating fields in high-grade glioma: a real-world retrospective analysis of 28 patients

**DOI:** 10.3389/fneur.2026.1771675

**Published:** 2026-06-24

**Authors:** Zichao Xu, Hongli Yang, Jiezhou Zheng, Zihuang Li, Mengqi Sun

**Affiliations:** Department of Radiation Oncology, Shenzhen People’s Hospital, The Second Clinical Medical College, Jinan University, Shenzhen, China

**Keywords:** chemoradiotherapy, efficacy analysis, high-grade glioma, multimodal therapy, tumor treating fields

## Abstract

**Objective:**

To evaluate the efficacy of Tumor Treating Fields (TTFields) in 28 patients with high-grade gliomas in a real-world setting.

**Methods:**

A retrospective analysis was performed on 19 newly diagnosed and 9 recurrent high-grade glioma patients treated at Shenzhen People’s Hospital from March 2019 to February 2022. All patients underwent TTFields therapy for at least one month. Tumor stability or progression was assessed using the Response Assessment in Neuro-Oncology (RANO) criteria. Progression-free survival (PFS) and overall survival (OS) were calculated with the Kaplan–Meier method. Adverse events were recorded using the Common Terminology Criteria for Adverse Events (CTCAE) version 5.0 and TTFields-related dermatologic adverse event (dAE) grading scale. Treatment adherence was assessed through the device usage data from the NonoTTF-200A system.

**Results:**

As of August 2024, the median duration of TTFields use was 9.4 months, with a median adherence rate of 88.05% (range: 54.3–92%). Among newly diagnosed patients, the median PFS and OS were 18.1 and 22.0 months, respectively. In the recurrent cohort, median PFS and OS were 9.1 and 18.2 months. The most common adverse events were grade 1–2 dermatologic reactions, including folliculitis, rash, and ulcers, with no grade 3–4 TTFields-related toxicities observed.

**Conclusion:**

These real-world, retrospective findings suggest that TTFields therapy is feasible, well tolerated, and associated with high treatment adherence in patients with high-grade gliomas. Observed survival outcomes were descriptively consistent with previously reported clinical and real-world data; however, given the small sample size and observational design, these findings should be interpreted cautiously. Prospective studies with larger cohorts are warranted to further evaluate the role of TTFields in glioma management.

## 1 Introduction

High-grade gliomas (HGGs) are the most common primary malignant tumors of the central nervous system, characterized by aggressive proliferation and invasiveness. Among these, glioblastoma (GBM) has the poorest prognosis. The current standard treatment for GBM involves maximal safe surgical resection, followed by adjuvant radiotherapy and concurrent maintenance chemotherapy with temozolomide (TMZ) (1). Despite this multimodal approach, the median progression-free survival (PFS) for GBM patients remains only 6.9 months, with a median overall survival (OS) of approximately 12.5–15 months. The approximately 2-year and 5-year survival rates are around 25 and 10%, respectively (2).

Tumor Treating Fields (TTFields) is a novel physical modality that has emerged over the past decade, utilizing low-intensity, intermediate-frequency alternating electric fields (100–300 kHz) to disrupt tumor cell division. Several landmark trials have evaluated the role of TTFields in GBM therapy. Notably, the phase III EF-14 trial, published in *JAMA* in 2015 and updated in 2017, demonstrated that adding TTFields to maintenance TMZ following radiochemotherapy significantly improved median OS in newly diagnosed GBM patients, from 16.0 months to 20.9 months. In addition, PFS was also significantly improved, establishing TTFields as part of the standard of care for newly diagnosed GBM. Earlier, the EF-11 trial assessed TTFields in recurrent GBM, confirming its safety and tolerability, though without significant survival advantage over chemotherapy (3, 4). TTFields was granted FDA approval for the treatment of recurrent and newly diagnosed GBM in adults in 2011 and 2015, respectively. Since its introduction in the United States in 2011, TTFields has been used to treat tens of thousands of glioma patients worldwide (5).

However, the two pivotal phase III clinical trials evaluating TTFields excluded Chinese GBM patients. In 2018, the Chinese National Health Commission’s guidelines for glioma diagnosis and treatment recommended TTFields for newly diagnosed GBM (Level 1 evidence) and recurrent HGGs (Level 2 evidence). Despite this, it wasn’t until May 2020 that TTFields received official approval for use in mainland China.

This study retrospectively analyzes clinical data from HGGs patients treated with TTFields at Shenzhen People’s Hospital from March 2019 to January 2022. Clinical follow-up was conducted until August 24, 2024. The goal is to report preliminary findings from a Chinese HGGs cohort treated with TTFields, offering insights that may inform future prospective research.

## 2 Materials and methods

This retrospective study included patients with histopathologically confirmed high-grade gliomas treated at the Department of Oncology and Radiotherapy, Shenzhen People’s Hospital, from March 2019 to January 2022. Eligible patients were identified through electronic chart review. Clinical follow-up was conducted until August 24, 2024, which served as the data cut-off date for survival analysis. Of the 30 patients screened, 2 were excluded due to incomplete data or loss to follow-up within 3 months, and 28 patients were included in the final cohort.

Given that all data cohorts were collected prior to the publication of the 2021 WHO Classification of Tumors of the Central Nervous System (CNS)—which redefined glioblastoma as IDH-wildtype and introduced astrocytoma, IDH-mutant, CNS WHO grade 4 as a separate entity— patients in this study were diagnosed according to the WHO 2016 classification. Under the WHO 2016 system, both glioblastoma, IDH-wildtype and glioblastoma, IDH-mutant were recognized as subtypes of glioblastoma. Therefore, a small number of patients with IDH-mutant WHO grade IV astrocytoma were included and treated following the glioblastoma management protocols applicable at that time. This classification approach reflects the clinical diagnostic and therapeutic standards used during the study period (2019–2022) and aligns with real-world practice before the WHO 2021 update.

Inclusion criteria included: (1) histopathologically confirmed high-grade glioma (WHO grade III–IV) according to the 2016 WHO classification; (2) treatment with standard concurrent chemoradiotherapy based on the Stupp protocol followed by TTFields for at least one month; (3) ability to undergo regular MRI follow-up; and (4) provision of written informed consent.

Exclusion criteria were infratentorial tumor location or leptomeningeal involvement, pregnancy or breastfeeding, concurrent enrollment in other clinical trials, and clinical signs of elevated intracranial pressure.

Treatment details: All newly diagnosed patients received the standard Stupp protocol, consisting of radiotherapy (60 Gy in 30 fractions) with concurrent temozolomide (75 mg/m^2^ daily), followed by adjuvant temozolomide (150–200 mg/m^2^, days 1–5 of a 28-day cycle) combined with TTFields. All recurrent patients received TTFields in combination with bevacizumab and temozolomide.

### 2.1 Patient information

All patients underwent TTFields therapy for at least one month. Among the 28 patients, 26 were diagnosed with GBM (Grade IV glioma), and 1 patient was diagnosed with gliosarcoma, a rare histological variant of GBM. The remaining patient had an initial diagnosis of oligodendroglioma but experienced rapid progression and death after recurrence. Although pathology was not reconfirmed at recurrence, this patient was clinically considered to have transformed into high-grade glioma. Baseline demographic and molecular characteristics are summarized in Table 1.

**Table 1:** General characteristics of 28 patients.

Characteristics	Newly diagnosed	Recurrent
Age (year)		
Mean ± SD	42.11 ± 17.18	51.33 ± 11.77
M (Q1-Q3)	40 (29, 56)	53 (47, 60)
Min–Max	15–68	29–65
Gender		
Male	10 (52.63%)	4 (44.44%)
Female	9 (47.37%)	5 (55.56%)
BASELINE_KPS		
Mean ± SD	75.79 ± 20.9	70 ± 21.21
M (Q1-Q3)	90 (60, 90)	80 (50, 90)
Min–Max	40–100	40–90
WHO grade		
WHO Grade 3	0 (0%)	1 (11.11%)
WHO Grade 4 (Recurrent)	0 (0%)	8 (88.89%)
WHO Grade 4 (New)	19 (100%)	0 (0%)
IDH status		
Wild type	17 (89.47%)	8 (88.89%)
Mutant	2 (10.53%)	1 (11.11%)
Unknown*	0 (0%)	0 (0%)
Extent of resection		
Gross total resection	12 (63.16%)	7 (77.78%)
Partial resection	7 (36.84%)	2 (22.22%)
MGMT promoter methylation		
Unmethylated	7 (36.84%)	2 (22.22%)
Methylated	7 (36.84%)	5 (55.56%)
Unknown*	5 (26.32%)	2 (22.22%)
1p/19q Codeletion		
Non-codeleted	18 (94.74%)	6 (66.67%)
Codeleted	0 (0%)	0 (0%)
Unknown*	1 (5.26%)	3 (33.33%)
TERT promoter		
Wild Type	8 (42.11%)	3 (33.33%)
Mutant	5 (26.32%)	4 (44.44%)
Unknown*	6 (31.58%)	2 (22.22%)
BRAF V600E		
Wild Type	13 (68.42%)	7 (77.78%)
Mutant	3 (15.79%)	0 (0%)
Unknown*	3 (15.79%)	2 (22.22%)
PTEN mutation		
No	4 (21.05%)	1 (11.11%)
Yes	1 (5.26%)	0 (0%)
Unknown*	14 (73.68%)	8 (88.89%)
Concomitant chemoradiotherapy		
No	7 (36.84%)	8 (88.89%)
Yes	12 (63.16%)	1 (11.11%)

^*^Unknown: indicates that comprehensive molecular testing was not performed due to financial constraints related to out-of-pocket payment.

Regarding molecular pathology, next-generation sequencing (NGS) was used to assess IDH status, TERT promoter mutation, EGFR amplification, chromosome 7 gain/chromosome 10 loss, PTEN (phosphatase and tensin homolog) mutation, and other relevant markers when available. Among the patients with sequencing results, 24 of 26 had IDH wild-type tumors (85.71%), 9 of 22 had unmethylated MGMT promoters (40.91%), and 9 of 19 had PTEN mutations (47.37%). Due to financial constraints associated with out-of-pocket payment for molecular testing, not all patients underwent comprehensive genomic profiling. The number of patients tested for each marker is indicated in Table 1, and the remaining were categorized as unknown.

### 2.2 TTFields method

The NovoTTF-200A device, manufactured by Novocure (Israel), was used to deliver low-intensity (1–3 V/cm), intermediate-frequency (200 kHz) alternating electric fields to the tumor site. Patients received TTFields therapy through two pairs of transducer arrays applied to the scalp.

The treatment protocol included the following steps: (1) Patient Selection: The physician assessed the patient’s eligibility for TTFields therapy, which was indicated for adult patients with supratentorial high-grade gliomas. Upon agreement, the patient signed the informed consent form. (2) MRI Preparation: Preoperative and recent (within one month) contrast-enhanced T1-weighted cranial MRI images (DICOM format, including axial and coronal views) were obtained. (3) Treatment Planning: A certified imaging technician utilized the “TTFields Planning System” software to analyze the MRI data, measuring head size, tumor location, and tumor size. The system then calculated the optimal placement of the transducer arrays and generated a schematic diagram.(4) Device Dispensation and Training: The patient visited the pharmacy to purchase the device and receive the transducer arrays, followed by training on device usage and its initial application.(5) Follow-up and Monitoring: The physician established an outpatient follow-up plan, including regular evaluations of therapeutic efficacy and potential adverse reactions.(6) Compliance Monitoring and Definition of Adherence: Compliance with TTFields therapy was monitored by a Device Support Specialist (DSS) using device-generated usage reports. In this real-world cohort, adherence was assessed based on treatment days. Specifically, adherence was defined as the proportion of days during which the patient used the TTFields device (usage days) relative to the total number of days from TTFields initiation to treatment discontinuation or last follow-up, expressed as a percentage: adherence (%) = (number of TTFields usage days / total number of treatment days) × 100. (7) Usage Guidelines: Patients were instructed to wear the device for a target of at least 18 h per day. Short breaks during daily treatment and longer interruptions (2–3 times per week) were allowed according to patient tolerance and clinical circumstances.

### 2.3 Efficacy and adverse event assessment

Following the initiation of TTFields treatment, contrast-enhanced cranial MRI was performed every two months or more frequently if there was clinical suspicion of tumor progression. Tumor stability or progression was assessed according to the Response Assessment in Neuro-Oncology (RANO) criteria (6). PFS and OS were analyzed using the Kaplan–Meier method.

Adverse events (AEs) were recorded according to the Common Terminology Criteria for Adverse Events (CTCAE) version 5.0 and the grading criteria specific to TTFields-related dermatologic adverse events (dAEs) (7). Dermatologic AEs were assessed through direct observation by the researchers or by reviewing the patient’s scalp photographs at least once a month. The severity of dAEs was graded as follows: Grade 1 (Mild/Asymptomatic): No intervention required or only local treatment necessary. Treatment interruption may be less than 3 days. Grade 2 (Moderate): Requires systemic treatment or treatment interruption for more than 3 days. Grade 3 (Severe): Not immediately life-threatening. Grade 4 (Life-Threatening): Requires urgent intervention.

### 2.4 Statistical methods

Data analysis was performed using SPSS version 26.0. Non-normally distributed quantitative data are presented as median (range) and were compared using the Mann–Whitney U test. Survival curves were generated using the Kaplan–Meier method. A *p*-value of <0.05 was considered statistically significant. Sensitivity analyses were performed by excluding the non-GBM patient to assess the robustness of the survival analyses.

## 3 Results

The median time from diagnosis to the initiation of TTFields for the 19 newly diagnosed patients was 11.9 weeks (95% CI,7.9–16.0 weeks). Among these 19 patients, 12 underwent total resection, while 7 underwent partial resection. And 12 patients began TTFields therapy during concurrent radiotherapy, with daily changes to the transducer arrays during chemoradiotherapy. 7 patients started TTFields after completing concurrent chemoradiotherapy as part of adjuvant therapy.

For the 9 patients with recurrent HGGs, TTFields was initiated after recurrence was confirmed. The median time from confirmation of recurrence to the initiation of TTFields was 6.2 weeks (95% CI, 6.1–6.3 weeks).

### 3.1 Treatment duration and compliance

The median treatment duration for the 28 patients was 9.4 months (95% CI, 7.0–11.9 months). Based on a usage-day definition, the median adherence rate was 88.05% (95% CI, 54.3–92%) in the overall cohort. Among newly diagnosed patients, the median adherence rate was 89.45% (95% CI, 82–92%), while in recurrent patients it was 86.35% (95% CI, 54.3–91.8%).

### 3.2 Follow-up and patient survival

**Figure 1:**
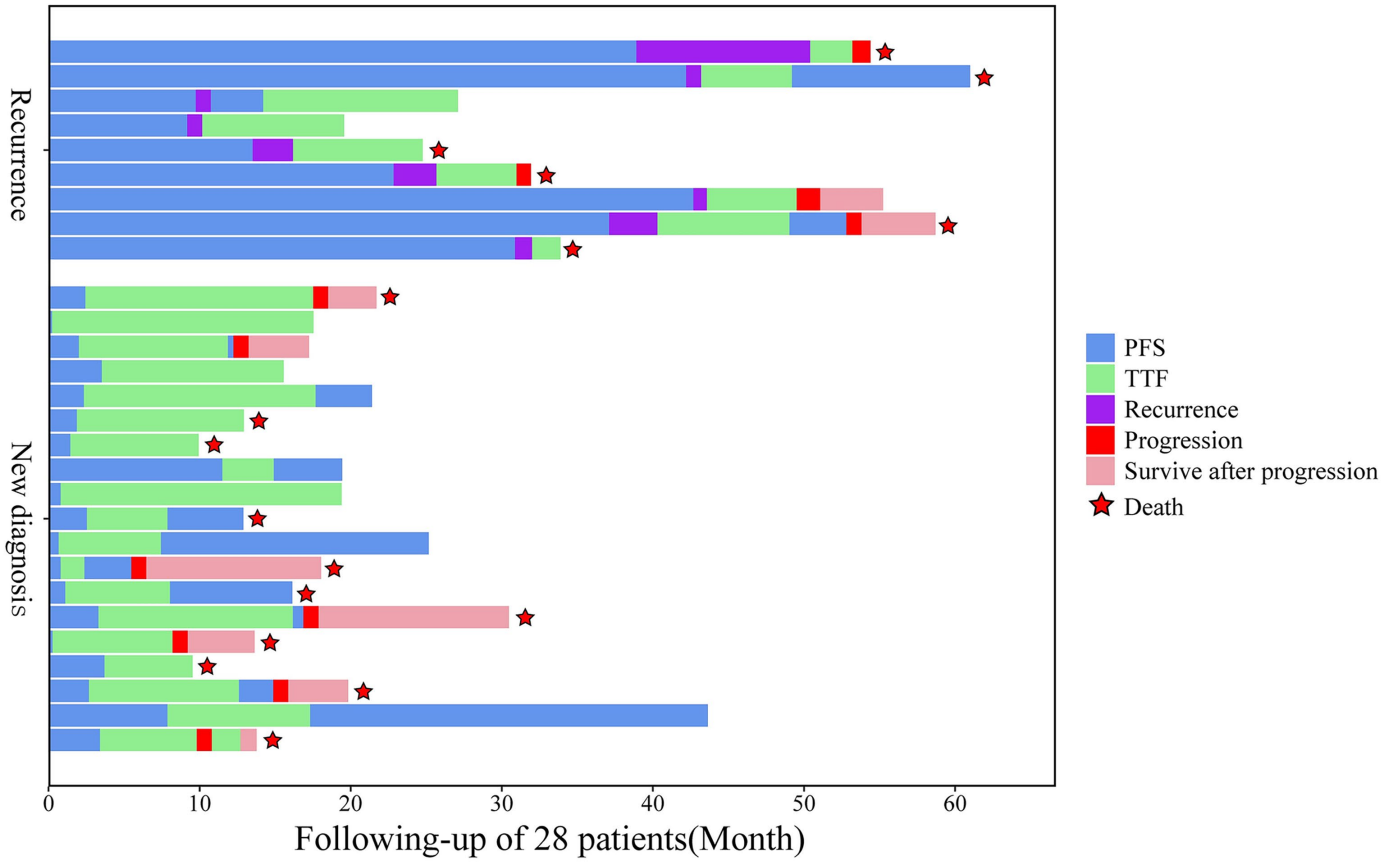
Follow-up of 28 patients.

**Figure 2:**
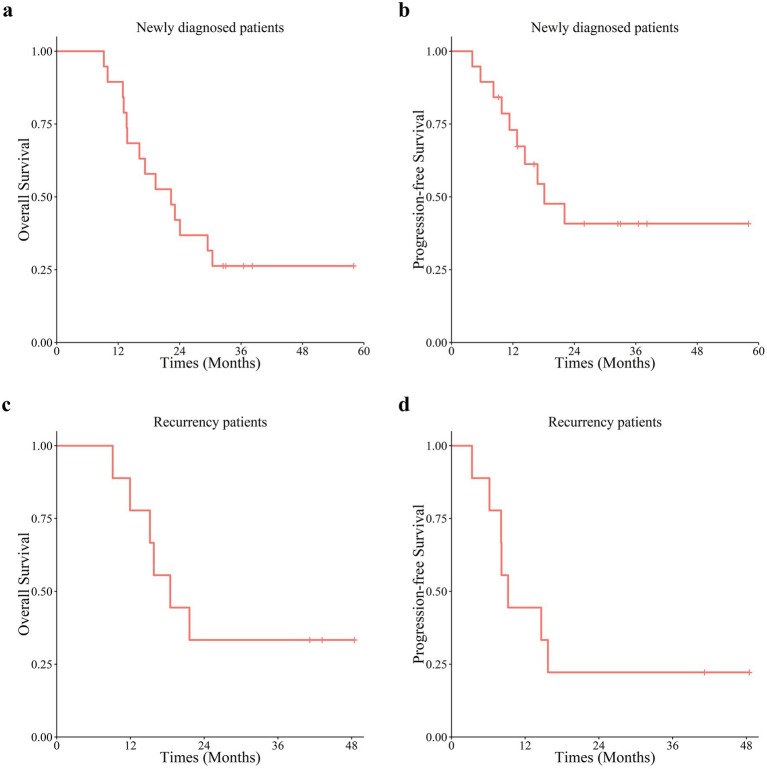
Kaplan–Meier survival curves for patients treated with TTFields; **(a, b)** OS and PFS curve for 19 newly diagnosed HGGs patients; **(c, d)** OS and PFS curve for 9 recurrent HGGs patients. Tick marks indicate censored observations. Survival curves were estimated using the Kaplan–Meier method. Comparisons between newly diagnosed and recurrent patients showed no statistically significant differences in OS (log-rank *p* = 0.898) or PFS (log-rank *p* = 0.412). Corresponding number-at-risk tables are provided in Supplementary Tables S1, S2.

As of August 2024, of the 19 newly diagnosed patients, 5 were alive at the last follow-up, and 14 had died (Figure 1). The median PFS was 18.1 months (95% CI, 12.0–24.2 months) (Figure 2a), and the median OS was 22.0 months (95% CI, 13.9–30.1 months) (Figure 2b). Among the 9 recurrent patients, 2 remained stable, 1 progressed, and 6 died (Figure 1). The median PFS after recurrence was 9.1 months (95% CI, 5.9–12.3 months) (Figure 2c), and the median OS was 18.2 months (95% CI, 10.6–25.8 months) (Figure 2d). Kaplan–Meier analysis showed no statistically significant difference in OS between newly diagnosed and recurrent patients (log-rank *p* = 0.898). Similarly, no significant difference in PFS was observed between the two groups (log-rank *p* = 0.543). The sensitivity analysis excluding the non-GBM patient yielded similar survival trends and did not materially alter the overall results. Univariable Cox proportional hazards analyses for PFS and OS are summarized in Table 2. None of the evaluated clinical or molecular variables, including age, sex, Karnofsky performance status (KPS), IDH status, extent of resection, MGMT promoter methylation, or concomitant chemoradiotherapy, showed a statistically significant association with PFS or OS.

**Table 2:** Univariable Cox proportional hazards analysis for PFS and OS.

Variable	PFS		OS	
	HR (95% CI)	*p*-value	HR (95% CI)	*p*-value
Age (years): Median (Min, Max)	1.02 (0.99,1.04)	0.211	1.00 (0.98, 1.03)	0.812
Sex: Male vs. Female	0.70 (0.31,1.56)	0.381	0.66 (0.28, 1.58)	0.351
KPS: Median (Min, Max)	0.99 (0.98,1.01)	0.530	1.00 (0.98, 1.02)	0.700
IDH Status: Wild type vs. Mutant	0.54 (0.22,1.35)	0.187	0.43 (0.15, 1.29)	0.134
Extent of resection: GTR vs. PR	1.27 (0.54,2.96)	0.587	1.35 (0.54, 3.35)	0.525
MGMT: Meth vs. Unmeth	0.93 (0.52,1.67)	0.817	1.22 (0.68,2.19)	0.499
Concomitant Chemoradiotherapy: Yes vs. No	0.86 (0.38,1.96)	0.725	0.78 (0.32, 1.89)	0.587

When stratified by disease status, the type and frequency of TTFields-related adverse events were similar between newly diagnosed and recurrent patients. No patients experienced grade 3–4 dAEs. Among the 28 patients, 22 experienced grade 1 dAEs (78.6%, 22/28), and 6 experienced grade 2 dAEs (21.4%, 6/28). The most common symptoms included allergic dermatitis (85.7%, 24/28), erythema and pruritus (67.8%, 19/28), folliculitis (17.9%, 5/28), blisters (10.7%, 3/28), and ulcers (7.1%, 2/28). Three patients required treatment interruption due to scalp-related adverse effects. After repositioning the transducer arrays and applying corticosteroid ointment, symptoms were alleviated. The adverse events observed were mild and manageable, and no patients required permanent discontinuation of TTFields due to toxicity, indicating good tolerability in this real-world cohort.

## 4 Discussion

GBM is the most common primary malignant tumor of the central nervous system in adults and is known for its high malignancy. Patients with GBM who undergo surgery followed by the Stupp regimen for adjuvant treatment have an OS of only 10–16 months, with a 5-year survival rate still under 10% (1, 8). Clinical advances in GBM treatment have been slow over the past few decades. However, TTFields have emerged as a novel physical treatment modality in recent years. TTFields work by applying low-frequency alternating electric fields to interfere with the mitotic process of tumor cells, inhibiting their growth. Additionally, TTFields have tumor-suppressive effects through mechanisms such as interference with DNA repair, modulation of cell permeability, and enhancement of immune responses (3, 9–11).

The EF-11 and EF-14 studies are two pivotal phase III randomized controlled trials evaluating the safety and efficacy of TTFields. The EF-11 study demonstrated that in recurrent GBM patients, TTFields monotherapy did not significantly improve median PFS (2.2 months vs. 2.1 months) or OS (6.6 months vs. 6.0 months) compared to palliative chemotherapy. However, TTFields resulted in fewer adverse events and improved quality of life (12). The EF-14 study, which involved newly diagnosed GBM patients, showed that compared to TMZ monotherapy, the addition of TTFields to TMZ as adjuvant therapy extended median PFS from 4.0 months to 6.7 months and median OS from 16.0 months to 20.9 months, with no increase in systemic adverse events and no significant decline in quality of life (12). A real-world retrospective study also reported a median OS of 9.6 months for 457 recurrent GBM patients treated with TTFields (5).

In our study, 19 newly diagnosed HGGs patients were included, among whom 18 were diagnosed with glioblastoma. As of August 24, 2024, five patients remained alive at the last follow-up, while fourteen had died. Notably, one patient died from brainstem hemorrhage despite having radiographically stable disease. The median OS of 22.0 months observed in newly diagnosed patients in our cohort is consistent with that reported in the pivotal EF-14 trial (20.9 months). In addition, our cohort demonstrated generally high adherence to TTFields therapy, which is encouraging given the real-world challenges associated with device use and patient compliance. Prior evidence, including the EF-14 trial, suggests that achieving ≥75% adherence is associated with improved survival outcomes. Together, these findings support the external consistency and real-world feasibility of TTFields therapy, although direct statistical comparisons with randomized trials were not feasible due to the limited sample size and single-center design. In univariable Cox proportional hazards analyses, no clinical or molecular factors were significantly associated with PFS or OS. This is likely attributable to the limited cohort size, small number of events, and heterogeneity inherent to this retrospective real-world study.

In this study, 25% (7/28) of patients were elderly, 25% (7/28) had a KPS of less than 60, and 32.1% (9/28) had multiple lesions or residual tumor after surgery. As a result, some patients received individualized treatment regimens. The SPARE study has already validated the safety of combining TTFields with concurrent chemoradiotherapy (13, 14), and in this study, 12 newly diagnosed HGGs patients received TTFields during concurrent chemoradiotherapy. Among these 12 patients, one received increased single-dose radiation (60Gy/20 fractions) to the residual tumor following partial resection due to a significant post-surgical tumor burden. This patient was also treated with TTFields, BEV, and TMZ. Imaging follow-up confirmed tumor control, but the patient later died from distant recurrence and metastasis. Another patient, who had a poor postoperative condition, received a lower-dose radiation regimen (40.05Gy/15 fractions). Imaging follow-up revealed both primary tumor and distant metastasis, and the patient was treated with TTFields combined with TMZ chemotherapy. By the study’s cutoff date, the patient’s condition remained stable, with a total OS of 50 months. These individualized treatment regimens likely influenced the survival outcomes in this cohort.

Previous studies have suggested that molecular alterations may influence outcomes in glioblastoma patients treated with TTFields (15). For example, PTEN, a tumor suppressor gene frequently altered in human cancers, is mutated in approximately 40% of glioblastomas (16). Prior retrospective analyses have reported that recurrent GBM patients with PTEN mutations receiving TTFields experienced longer OS and PFS compared with those harboring wild-type PTEN (15). However, these observations were derived from independent cohorts and were not evaluated within the present study due to incomplete molecular profiling.

Another molecular alteration of interest is the BRAF V600E mutation, which occurs in approximately 1–2% of GBM cases (17, 18). Previous studies and case reports have demonstrated the safety and potential efficacy of BRAF V600E inhibitors, including in combination with TTFields, in selected patients with high-grade gliomas (19, 20). In our cohort, although three patients harbored BRAF V600E mutations, none received BRAF or MEK inhibitor therapy. Given the limited sample size and incomplete molecular data, no conclusions regarding the prognostic impact of BRAF mutations can be drawn from our study, and these findings are discussed solely in the context of existing literature.

In conclusion, while studies have confirmed the clinical benefits of TTFields in glioma treatment, research in this area is ongoing. The findings of this retrospective study provide preliminary, observational insights into the safety and feasibility of TTFields therapy in a small Chinese glioma cohort. And these observations may help inform future prospective investigations. Importantly, although some survival observations appeared consistent with prior randomized trials, such as EF-14, this retrospective analysis was not designed to establish efficacy or equivalence, and any such comparisons should be interpreted descriptively rather than inferentially.

However, this study has several limitations. First, this was a retrospective, single-center analysis with a relatively small sample size, which may introduce selection bias and limit the generalizability of the findings. In addition, the absence of a randomized control group precludes definitive conclusions regarding the therapeutic efficacy of TTFields. Second, the extent of surgical resection is a well-established prognostic factor in GBM. In our study, 12 patients underwent gross total resection, while 7 underwent partial resection. Although this factor may have influenced prognosis, subgroup numbers were too small for further stratified analysis. For recurrent patients, heterogeneity in prior surgical management also represented a limitation. These variables should be considered when interpreting the TTFields outcomes. Third, the inclusion of one oligodendroglioma patient—although justified by clinical course—may introduce heterogeneity into the study population. Lastly, although all tumors in our cohort were located in the supratentorial region, it is important to recognize that tumor depth and proximity to eloquent cortical areas may affect prognosis and treatment response. Due to the small sample size, we were unable to perform a meaningful subgroup analysis by tumor location. Future prospective studies with larger cohorts are warranted to further explore this factor. Future research from our center is already underway and aims to address these limitations by enrolling larger, more molecularly-defined cohorts, conducting prospective analyses, and exploring predictive biomarkers for TTFields responsiveness to support more individualized treatment strategies.

## Data Availability

The datasets generated and analyzed during the current study are not publicly available due to patient privacy and institutional policy, but are available from the corresponding author upon reasonable request. Requests to access these datasets should be directed to Mengqi Sun: sunmengqi1990@126.com.
