# Supplementary material for: Efficacy of tumor treating fields in high-grade glioma: a real-world retrospective analysis of 28 patients
Source: Front Neurol. 2026 Jun 24;17:1771675. doi: 10.3389/fneur.2026.1771675 (PMC13341559; doi:10.3389/fneur.2026.1771675)
Supplement: Supplementary file 1 [file Table_1.DOCX]

Supplementary Table 1Number at risk for overall survival

| Months | 0 | 12 | 24 | 36 | 48 | 60 |
| --- | --- | --- | --- | --- | --- | --- |
| Newly diagnosed | 19 | 17 | 7 | 2 | 1 | 0 |
| Recurrent | 9 | 7 | 3 | 2 | 0 | 0 |

Supplementary Table 2 Number at risk for progression-free survival

| Months | 0 | 12 | 24 | 36 | 48 | 60 |
| --- | --- | --- | --- | --- | --- | --- |
| Newly diagnosed | 19 | 13 | 3 | 1 | 1 | 0 |
| Recurrent | 9 | 3 | 2 | 2 | 0 | 0 |
